# B7-H6/NKp30 Axis in Melanoma: Translational Rationale, Evidence Gaps, and Therapeutic Considerations

**DOI:** 10.3390/biom16060862

**Published:** 2026-06-12

**Authors:** Kevin M. Truong-Balderas, Rachel C. Chang, Claudia Lasalle, Yi Gao, Nicole C. Nowak, Kyle T. Amber, Adrian P. Mansini

**Affiliations:** 1 Department of Dermatology, Rush University Medical Center, Chicago, IL 60612, USA; kevin_m_truong-balderas@rush.edu (K.M.T.-B.); rachel_chang@rush.edu (R.C.C.); clasal3@uic.edu (C.L.); yi_gao@rush.edu (Y.G.); nicole_c_nowak@rush.edu (N.C.N.); kyle_amber@rush.edu (K.T.A.); 2 Department of Urology, Rush University Medical Center, Chicago, IL 60612, USA

**Keywords:** B7-H6, NKp30, melanoma, natural killer cells, immune surveillance, immune escape, bispecific engagers, CAR-T, soluble B7-H6

## Abstract

Melanoma treatment has been transformed by immune checkpoint blockade, yet many patients still experience primary resistance, limited durability of response, or acquired resistance. These limitations underscore the need for additional targets that reflect melanoma biology while enabling new therapeutic strategies, particularly in biologically defined settings of immune escape such as checkpoint-resistant, HLA-low, dedifferentiated, or stress-adapted melanoma. The B7-H6/NKp30 axis has gained attention as a link between tumor cell stress, immune recognition, and therapy-related adaptation. B7-H6 (*NCR3LG1*), an inducible ligand for NKp30, has been detected in melanoma cell lines and tumor specimens, and soluble B7-H6 has been identified in a subset of patients. Membrane-bound B7-H6 may support NK-cell activation, whereas ligand shedding and accumulation of soluble B7-H6 may reduce effective antitumor recognition and promote immune evasion. Emerging evidence further suggests that B7-H6 expression may be linked to tumor-intrinsic programs relevant to melanoma cell survival, migration, and adaptation to therapeutic stress. However, B7-H6 is not yet a validated predictive biomarker or an established therapeutic target in melanoma, and current evidence remains limited by small melanoma-specific datasets, incomplete information on spatial and temporal heterogeneity, and the absence of melanoma-focused clinical validation. In this review, we examine the role of the B7-H6/NKp30 axis in immune surveillance, tumor escape, biomarker development, and therapeutic targeting, and discuss its translational potential in melanoma as an emerging but incompletely validated pathway that warrants focused investigation in melanoma states where conventional immune control is limited.

## 1. Introduction

Melanoma remains one of the most immunologically dynamic solid tumors, yet durable disease control remains limited by primary resistance, incomplete response durability, or acquired resistance [1]. Although immune checkpoint blockade and MAPK-targeted therapy have improved outcomes in selected patients, their clinical benefit is frequently limited by non-response and eventual relapse, underscoring the need for additional targets that reflect melanoma biology and therapeutic vulnerability [1,2].

The B7-H6/NKp30 axis has emerged as a candidate pathway for such investigation. B7-H6, encoded by *NCR3LG1*, was originally identified as a tumor-associated ligand for the activating natural cytotoxicity receptor NKp30 and is notable for its minimal expression in most healthy tissues under normal conditions [3]. This pattern immediately suggested translational potential: a stress-inducible surface ligand preferentially associated with malignant cells offers both targeting potential and biomarker interest. Since its initial description, the pathway has expanded from a tumor-recognition mechanism to a broader framework that includes ligand shedding, stress-dependent regulation, receptor-based engineering, and T-cell–redirecting strategies [4,5,6,7].

### Rationale for Focusing on Melanoma

Although melanoma is among the solid tumors most responsive to immune checkpoint blockade, it remains a highly relevant setting in which to examine additional immune-recognition pathways. A substantial fraction of patients either do not respond or eventually develop acquired resistance, and resistant melanoma states are frequently associated with impaired antigen presentation, HLA class I downregulation, dedifferentiation, immune exclusion, and therapy-induced plasticity [1,2,8,9,10]. These features provide a rationale for examining innate immune-recognition pathways that may complement conventional T-cell-mediated immunity. In this context, the B7-H6/NKp30 axis is of interest not because it is already validated as a melanoma biomarker or therapeutic target, but because it links tumor-cell stress, NK-cell recognition, ligand shedding, and target accessibility. Therefore, melanoma provides a clinically relevant and biologically informative setting in which to evaluate whether this pathway has particular importance in checkpoint-resistant, HLA-low, dedifferentiated, or stress-adapted disease states.

Several observations support a focused, hypothesis-generating examination of this pathway in melanoma. First, NK-cell biology remains relevant in melanoma, particularly in disease states that may become less accessible to conventional T-cell-mediated pressure, including HLA-low, dedifferentiated, or therapy-adapted tumors [11,12,13]. Second, B7-H6 has been detected in melanoma cell lines, melanoma tumor specimens, and circulating serum samples from patients with melanoma, although the available melanoma-specific evidence remains limited in cohort size and does not yet define expression across large primary and metastatic melanoma datasets [7,14]. Third, the axis is compatible with several translational strategies, including biomarker development, engineered cell- and antibody-based targeting, and approaches aimed at increasing B7-H6 surface density or limiting proteolytic shedding [7,15,16]. However, these opportunities remain investigational in melanoma and require careful consideration of target density, soluble ligand, spatial heterogeneity, and disease context.

**Figure 1:**
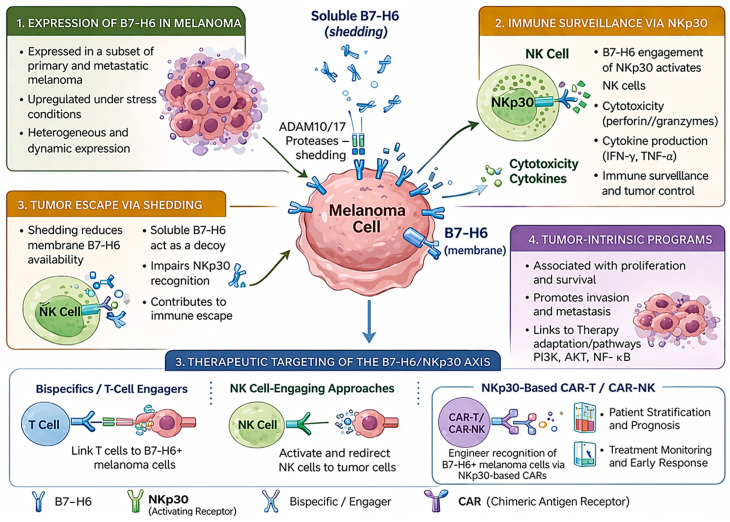
**Conceptual framework of the B7-H6/NKp30 axis in melanoma.** Membrane-bound B7-H6 on melanoma cells can promote NKp30-dependent immune recognition and NK-cell activation. Protease-mediated shedding reduces surface ligand availability and generates soluble B7-H6, a process that may contribute to immune escape and limit target accessibility. Emerging evidence also suggests that B7-H6 expression may be linked to tumor-intrinsic programs relevant to survival, invasion, and adaptation to therapeutic stress. **Abbreviations:** ADAM10/17, A disintegrin and metalloproteinase domain-containing proteins 10 and 17; B7-H6, B7 homolog 6; CAR-NK, chimeric antigen receptor natural killer cell; CAR-T, chimeric antigen receptor T-cell therapy; NK, natural killer; NKp30, natural cytotoxicity receptor 3; AKT, protein kinase B; IFN-γ, interferon gamma; NF-κB, nuclear factor kappa-light-chain-enhancer of activated B cells; PI3K, phosphoinositide 3-kinase; TNF-α, tumor necrosis factor alpha.

In this review, we examine the B7-H6/NKp30 axis in melanoma through four connected lenses: immune surveillance, tumor immune escape, tumor-intrinsic regulation, and therapeutic development. Rather than presenting B7-H6 as an established melanoma biomarker or validated therapeutic target, we critically evaluate the current evidence, define key limitations, and outline the melanoma disease states in which this pathway may warrant focused translational investigation. We argue that the B7-H6/NKp30 axis is best viewed as an emerging and incompletely validated pathway with potential relevance in checkpoint-resistant, HLA-low, dedifferentiated, or stress-adapted melanoma. Figure 1 summarizes this conceptual framework.

## 2. B7-H6/NKp30 Axis in Immune Surveillance in Melanoma

Natural killer cells are central mediators of innate antitumor immunity and may be especially important in tumors with impaired antigen presentation or T-cell escape [13,17]. NK cells detect transformed cells by integrating activating and inhibitory signals through the missing-self and induced-self mechanisms [18,19]. In melanoma, both processes are relevant, as tumor progression is frequently associated with reduced HLA class I expression, while many melanoma cells retain expression of stress-induced activating ligands that support NK-cell recognition [8,20]. In melanoma, NK cells contribute to immunosurveillance through direct cytotoxicity, cytokine production, and interaction with dendritic cells and adaptive immune populations. This may be especially important in therapy-adapted melanoma states with reduced visibility to conventional T-cell-mediated immunity [6,7].

NKp30 is a major activating receptor on human NK cells. Its interaction with B7-H6 provides a direct route for recognition of stressed or transformed cells [3,4,5]. Structural studies established the molecular basis of NKp30–B7-H6 engagement and showed that this receptor–ligand pair represents a dedicated activating interaction rather than an inhibitory checkpoint-like pathway [4,5]. Because the axis promotes immune activation rather than immune restraint, it offers a distinct route of tumor–immune recognition in melanoma [4,5].

In metastatic disease, altered NK-receptor programs and reduced NK-cell fitness have been associated with progression and outcome, while distinct *NCR3* (encoding NKp30) transcript patterns have been described in clinically defined melanoma subsets [21]. These findings do not, on their own, establish B7-H6 as a validated therapeutic target, but they indicate that the biology engaged by the pathway already has biologic importance in melanoma. In other words, this axis extends beyond target expression alone and includes the NK-cell circuitry that shapes disease evolution [21].

Notably, the functional significance of this axis in melanoma is shaped not only by ligand availability on tumor cells, but also by progressive dysfunction of the NK-cell compartment within the tumor microenvironment. Experimental co-culture studies have shown that melanoma cells can downregulate NKp30 on NK cells and reduce cytolytic activity. In parallel, melanoma-associated fibroblasts have been shown to suppress NK-cell cytotoxicity, inhibit IL-2-driven NKp30 upregulation, and impair acquisition of cytotoxic granules [22]. Together, these findings suggest that melanoma undermines NK-cell surveillance through both tumor-intrinsic and microenvironmental mechanisms, reinforcing the idea that the B7-H6/NKp30 axis should be viewed as a framework shaped by both target expression and effector-cell competence.

Thus, in melanoma, the B7-H6/NKp30 axis matters not only for immune surveillance but also for therapeutic design because it identifies a route of tumor recognition that may remain actionable even as other mechanisms of immune escape emerge.

## 3. Tumor Escape Mechanisms: Shedding, Soluble B7-H6, and Target Loss

Although the B7-H6/NKp30 interaction can promote immune recognition, melanoma cells are not passive participants in this process. A key escape mechanism is proteolytic shedding of B7-H6 from the tumor cell surface, primarily mediated by A disintegrin and metalloproteinase domain-containing protein 10 (ADAM10) and 17 (ADAM17) [7]. This process directly reduces the amount of membrane-bound ligand available for NKp30-dependent recognition while simultaneously generating a soluble form detectable in patient serum [7]. In this regard, detection of soluble B7-H6 in patients with melanoma provides not only evidence of clinical detectability, but also a reminder that target presence and target accessibility are not necessarily equivalent states.

Biologically, this is a direct mechanism of tumor adaptation. A surface ligand that would otherwise signal tumor stress and susceptibility to NK-cell attack is converted into an escape-associated state characterized by reduced membrane density and the release of soluble antigen. In melanoma, this matters because emerging surface-directed therapeutic strategies, including CAR-based cellular therapies and bispecific antibodies, rely on adequate target display at the tumor cell surface. Consistent with this principle, CAR-T cell activity is strongly influenced by membrane antigen density, and melanoma-directed CAR-T development has highlighted the importance of sufficient surface target expression for effective tumor-cell killing [23,24]. If B7-H6 is actively shed, then target positivity in tissue may not fully capture real-time therapeutic accessibility.

Soluble B7-H6 is not simply a passive cleavage product. Prior work indicates that extracellular B7-H6 can contribute to impaired NK-cell function and dysregulated NKp30-dependent responses [25]. For B7-H6-directed therapies, soluble B7-H6 may also create a practical pharmacologic barrier. Depending on concentration, binding affinity, and therapeutic format, soluble B7-H6 could act as a circulating or local antigen sink, reducing the amount of CAR-T cells, NKp30-based receptors, NK-cell engagers, or bispecific antibodies available to engage membrane-bound B7-H6 on melanoma cells. It may also promote ineffective receptor engagement or reduce functional avidity at the tumor-cell surface. Thus, a tumor that is transcriptionally or histologically B7-H6-positive may still be poorly suited for B7-H6-directed therapy if most of the ligand is shed rather than retained at the membrane. This suggests a two-part escape mechanism: melanoma cells may reduce direct surface recognition while also blunting effective effector responses. This interpretation is consistent with broader evidence that NK-cell dysfunction is a feature of melanoma in both experimental systems and patients, further supporting the view that shedding should be understood not only as a mechanism of target loss, but also as part of a wider process of effector-cell disengagement.

These findings have direct therapeutic implications. Membrane-bound and soluble B7-H6 should not be considered equivalent, because surface-retained ligand supports NKp30-dependent recognition, whereas soluble B7-H6 reflects shedding-associated target loss and may carry different biologic and therapeutic meaning [7,25]. Accordingly, development of B7-H6-directed therapies in melanoma will likely require more than baseline tissue staining alone. A more informative strategy would integrate membrane B7-H6 density, soluble B7-H6 levels, and spatial heterogeneity in surface retention, particularly when interpreting response, resistance, or eligibility for surface-directed therapies [7].

The shedding mechanism also raises the possibility of therapeutic intervention at the level of target retention. Pharmacologic inhibition of ADAM10/17 increased surface B7-H6 and enhanced NK-cell-mediated killing, suggesting that ligand stabilization may improve target accessibility [7]. However, broad ADAM10/17 inhibition should currently be viewed as a mechanistic tool rather than a clinically realistic melanoma strategy, given the broad biologic roles of metalloproteases, potential toxicity, and limited therapeutic specificity. More practical near-term approaches may include selecting patients or lesions with high membrane-bound B7-H6 and low soluble B7-H6, incorporating soluble B7-H6 as a pharmacodynamic or exclusionary biomarker, designing therapeutic binders less susceptible to soluble antigen interference, and developing combination strategies that increase surface retention without broadly disrupting ADAM10/17-dependent biology. Importantly, this shedding process may be more dynamic than a fixed constitutive event. The incomplete suppression of B7-H6 release after ADAM10/17 inhibition suggests that additional regulatory inputs influence membrane ligand retention [7]. In melanoma, these mechanisms remain incompletely defined and should currently be viewed as plausible rather than established.

## 4. Regulation and Tumor-Intrinsic Functions

B7-H6 expression can be shaped by cellular stress, inflammatory cues, oncogenic transcriptional programs, and post-translational processing, including shedding and glycosylation. This makes the pathway biologically rich, but also more nuanced to interpret. In melanoma, where therapy-driven state transitions and phenotypic plasticity are pervasive, B7-H6 may be better understood as a dynamic readout of tumor state than as a fixed antigenic label [7,9,16,26,27,28].

At the transcriptional level, one of the clearest regulatory mechanisms involves *c-Myc*. Textor and colleagues showed that Myc directly drives B7-H6 transcription and that Myc inhibition reduces B7-H6 expression and impairs NKp30-dependent NK-cell degranulation [26]. This links ligand availability to tumor-intrinsic oncogenic programming, suggesting that Myc-related perturbations may alter target visibility in clinically important settings.

Stress-pathway regulation further expands this concept. Across tumor systems, B7-H6 can be induced by genotoxic stressors such as chemotherapy and radiotherapy, by inflammatory cues including TNF-α, by non-lethal heat shock, and by activation of integrated stress response pathways [16,27]. In melanoma, this matters because advanced disease is shaped by chronic cellular stress, nutrient limitation, microenvironmental pressure, and the adaptive consequences of prior therapy. These conditions may induce B7-H6 expression and help define the melanoma cell states in which this axis becomes most prominent under stress or treatment pressure [9,16,27]. Additional support for this model comes from melanoma-relevant stress settings. Endoplasmic reticulum stress and integrated stress response signaling have been shown to increase B7-H6 surface expression in melanoma cell lines, raising the possibility that B7-H6 may mark stress-adapted tumor states rather than a static antigenic identity [16,29].

Beyond regulation, emerging evidence suggests that B7-H6 may have tumor-intrinsic roles. In cutaneous melanoma, B7-H6 silencing reduced survival, migration, and clonogenicity while increasing sensitivity to dacarbazine in A375 cells [14]. Although these findings remain limited and require validation across broader melanoma models, they are nonetheless informative. They suggest that B7-H6 expression may mark melanoma cells with distinct survival or adaptive properties [14]. In addition, B7-H6 knockdown combined with dacarbazine was associated with reduced STAT3 protein expression in A375 melanoma cells, suggesting that the tumor-intrinsic effects of B7-H6 in melanoma may also involve survival-linked signaling programs. However, this observation remains limited and is currently based on a single model.

Post-translational regulation may add another layer of complexity to this axis. N-linked glycosylation can influence both NKp30 binding and membrane stability, supporting the idea that glycan state may affect the balance between membrane-bound and soluble B7-H6. Functional studies have identified distinct glycosylation sites with separable roles, including N208, which appears to support membrane stability and whose loss is associated with increased shedding, while N43 primarily affects NKp30 binding affinity [28]. Although this has not yet been defined in melanoma, altered glycosylation is a common feature of malignancy and can influence the stability and proteolytic processing of immune surface proteins [30,31].

### Potential Off-Tumor Expression Under Inflammatory Stress

The stress-responsive nature of B7-H6 also raises an important safety consideration for therapeutic targeting. Although B7-H6 was originally described as a tumor-associated ligand with minimal expression in most healthy tissues under basal conditions [3], its inducibility by cellular stress, inflammatory cytokines, and integrated stress response signaling suggests that expression may not be restricted to malignant cells under all biologic contexts [16,27]. Inflammatory or injured non-malignant tissues could theoretically upregulate B7-H6 or related stress-associated programs, creating a potential risk of off-tumor recognition by B7-H6-directed CAR-based therapies, NK-cell engagers, or bispecific antibodies.

This possibility is particularly relevant in melanoma, where patients may have treatment-induced inflammation, immune-related adverse events, prior radiation, tissue injury, or chronic inflammatory comorbidities. At present, there is insufficient evidence to define the frequency, magnitude, or functional significance of inducible B7-H6 expression in inflamed non-malignant human tissues. Therefore, B7-H6-directed therapeutic development should include careful profiling of normal and inflamed tissues, assessment of membrane-bound versus soluble B7-H6, and evaluation of whether inflammatory states alter target accessibility or safety. These considerations do not preclude therapeutic development, but they reinforce the need to define a therapeutic window based on membrane target density, tumor selectivity, soluble ligand burden, and the inflammatory context in which B7-H6 is expressed.

## 5. Expression Landscape and Biomarker Opportunities

Evidence for B7-H6 expression in melanoma remains limited, but it is sufficient to justify translational interest. In the original description of the ligand, melanoma cell lines were included within the early tumor expression landscape of B7-H6 [3]. Beyond its restricted tumor-associated expression, the NKp30–B7-H6 axis is also mechanistically notable because it functions as an activating rather than inhibitory immune interaction. Engagement of NKp30 by B7-H6 promotes NK-cell activation, including cytotoxic degranulation and proinflammatory cytokine release. Unlike classical inhibitory checkpoint pathways, this interaction is structurally and functionally activating, with NKp30 engaging B7-H6 through both the front and back β-sheets of its IgV-like domain [4]. Together, these features support the NKp30–B7-H6 axis as a mechanistically distinct immunotherapeutic target and provide a strong rationale for examining this axis in melanoma. More direct melanoma-specific evidence later showed B7-H6 expression in melanoma specimens in situ and detected soluble B7-H6 in the serum of a subset of patients [7]. Taken together, available data indicate that B7-H6 expression in melanoma is detectable across experimental and clinical contexts, but heterogeneous in magnitude and form [32]. Because B7-H6 is inducible, this variability is not unexpected. However, it has clear translational implications, because therapeutic tractability depends less on transcript abundance alone than on whether B7-H6 is present in a therapeutically accessible surface form.

An important advance came from work showing that B7-H6 transcripts are detectable in primary melanoma samples, whereas surface expression is variable and does not necessarily correlate with transcript abundance, consistent with additional post-transcriptional regulation [32]. This has direct implications for therapeutic development. In melanoma, the key question is not simply whether B7-H6 is expressed, but whether it is expressed in a form and at a density that can support intervention.

Soluble B7-H6 may complement tissue-based profiling as a minimally invasive readout of ligand shedding and changes in target form, but its interpretation in melanoma should be approached cautiously [7]. In melanoma, where spatial heterogeneity and multiple metastatic sites often complicate tissue-based evaluation, a circulating assay could provide useful longitudinal information, but it is unlikely to reflect the full target landscape on its own. Circulating B7-H6 levels may be influenced not only by tumor burden and shedding activity, but also by melanoma plasticity, organ-specific metastatic microenvironments, systemic inflammation, metabolic state, comedications, prior therapy exposure, and treatment timing. Therefore, soluble B7-H6 should not currently be viewed as a stand-alone predictive biomarker or a surrogate for membrane-bound target accessibility. Instead, its most plausible near-term role is as a complementary pharmacodynamic or exploratory biomarker that should be interpreted together with tissue B7-H6 expression, membrane target density, spatial distribution, and clinical context [7,9,33].

At the same time, biomarker development for this axis should be approached cautiously. Membrane-bound and soluble B7-H6 likely capture different aspects of tumor biology and therapeutic accessibility [7,34]. A tumor with abundant soluble B7-H6 but limited membrane retention may still be biologically informative, yet less amenable to surface-directed intervention than one with stable cell-surface expression. Future biomarker strategies should therefore move beyond binary positivity and incorporate target density, target form, timing, and, where feasible, spatial distribution. Melanoma-centered evidence supporting this axis is summarized in Table 1 and is presented as clinical and preclinical evidence, separated to highlight the current strengths and limitations of melanoma-specific data.

### Limitations of the Current Evidence in Melanoma

Despite the biologic rationale for studying the B7-H6/NKp30 axis in melanoma, the current evidence base remains limited and should be interpreted cautiously. Most melanoma-specific data derive from a small number of studies, with limited numbers of melanoma cell lines, tumor specimens, and patient serum samples [7,32]. As a result, the prevalence, magnitude, and clinical significance of B7-H6 expression across primary melanoma, metastatic melanoma, and therapy-resistant disease remain incompletely defined. In particular, large, well-annotated melanoma cohorts are still needed to determine whether B7-H6 expression differs by disease stage, metastatic site, prior therapy exposure, HLA class I status, dedifferentiation state, or response to immune checkpoint blockade.

Another major limitation is the lack of spatial and temporal information. Available studies do not yet define whether B7-H6 expression is homogeneous or heterogeneous within individual lesions, whether it differs across metastatic sites within the same patient, or whether membrane-bound and soluble B7-H6 change dynamically during treatment. This is especially important in melanoma, where tumor plasticity, immune editing, organ-specific microenvironments, and therapy-induced state transitions can substantially alter antigen expression and immune vulnerability over time [9,33]. Therefore, baseline detection of B7-H6 should not be assumed to reflect durable target accessibility.

Therapeutic evidence is also preliminary. Although B7-H6-directed CAR-based, bispecific, NK-engaging, and cytokine-augmented approaches provide proof-of-concept for druggability of the axis, most therapeutic data are preclinical and many are not melanoma-specific [15,32,35]. To date, B7-H6 has not been established as a predictive biomarker or validated therapeutic target in melanoma, and there is no definitive clinical evidence that selecting patients based on B7-H6 expression improves outcome. Future studies should therefore distinguish clearly between melanoma-specific evidence, evidence extrapolated from other tumor types, and broader mechanistic studies of B7-H6/NKp30 biology.

Finally, the functional meaning of B7-H6 expression in melanoma remains unresolved. Membrane-bound B7-H6 may support NKp30-dependent recognition, whereas soluble B7-H6 may reflect shedding-associated target loss and may interfere with effective immune recognition or surface-directed therapies [7,25]. Thus, B7-H6 positivity alone is unlikely to be sufficient for therapeutic stratification. More informative approaches will likely require integrated assessment of membrane target density, soluble B7-H6, spatial distribution, NK-cell competence, ADAM10/17-mediated shedding, and the inflammatory or treatment context in which B7-H6 is expressed. These limitations do not negate the relevance of the pathway, but they define the level of evidence needed before B7-H6/NKp30 can be advanced as a melanoma biomarker or therapeutic target.

## 6. Therapeutic Targeting of the B7-H6/NKp30 Axis

In melanoma, this axis is attractive because B7-H6 is relatively tumor-restricted, biologically relevant, and compatible with multiple therapeutic platforms. Together, these features support the development of B7-H6-directed strategies across several modalities, including receptor-based cellular therapies, T-cell engagers, cytokine-augmented dual-engagement approaches, and strategies aimed at increasing surface target availability by modulating target density or limiting ligand shedding [3,7,15,32,35,36,37,38,39]. The main therapeutic frameworks and their melanoma-specific development considerations are summarized in Table 2. The table shows preclinical and clinical-development strategies to clarify their melanoma-specific relevance and current level of validation.

The sections below highlight the platforms most pertinent to melanoma translation and emphasize how target form, target density, and treatment context may influence therapeutic applicability. Because B7-H6 can be assessed in both membrane-bound and soluble forms, this axis may also support biomarker-guided development strategies that integrate tissue- and circulating-based readouts rather than relying on a single pretreatment assay.

### 6.1. T-Cell Redirection and Bispecific Engagers

B7-H6-directed bispecific engagers are among the most advanced therapeutic strategies in this space. These constructs recruit T cells to B7-H6-expressing tumor cells independently of conventional peptide–MHC recognition, a feature that may be especially useful in melanoma, where resistant lesions can exhibit impaired antigen presentation, HLA class I downregulation, and dedifferentiated cell states [10,37]. Preclinical studies have shown that B7-H6-specific bispecific T-cell engagers can induce potent tumor clearance and promote host antitumor immunity [37]. In melanoma, where the endogenous immune response may be present but ineffective, this strategy could help restore antitumor pressure, even in lesions that have already been adapted to evade checkpoint-based therapies.

The B7-H6/CD3-engaging BI 765049 further reinforces the tractability of the target by demonstrating that industrial and early clinical development of this pathway are feasible [15]. Although most public data are not melanoma-specific, the existence of a clinically developed B7-H6-directed engager supports the broader proposition that this axis is druggable in a therapeutically meaningful way.

### 6.2. NK Cell–Engaging Approaches

Because B7-H6 is the natural activating ligand for NKp30, NK cell-oriented strategies have especially strong biologic grounding. Affinity-matured B7-H6-based bispecific immunoligands that engage NKp30 have shown enhanced NK-cell-mediated tumor-cell lysis and increased proinflammatory cytokine release [38]. Such formats may be particularly useful in melanoma lesions with modest B7-H6 surface expression, where native receptor–ligand affinity could otherwise limit therapeutic performance. By increasing the functional efficiency of the interaction, these agents may broaden the range of target-positive disease states that can be therapeutically exploited.

More recent dual-engagement approaches have extended this concept by combining B7-H6-targeted bispecific antibodies with IL-15/IL-15Rα signaling, thereby enhancing both NK-cell- and T-cell-mediated activity in chemo-resistant solid-tumor models [35]. An important feature of this design is that IL-15 activity was tumor-anchored through B7-H6-directed delivery, offering a way to enhance local effector-cell activation while reducing the limitations of unrestricted cytokine exposure [35]. In that study, NK-cell-engaging formats combined with localized IL-15 showed particularly strong activity, further supporting NK-centered, cytokine-augmented strategies as a promising direction for resistant solid tumors.

### 6.3. NKp30-Based CAR Therapies

NKp30-based CARs use the physiologic receptor domain to recognize B7-H6-positive tumor cells, thereby preserving a direct conceptual link between endogenous immune surveillance and engineered cellular therapy [36]. This is especially appealing in melanoma, where therapeutic success increasingly depends on identifying vulnerabilities that remain accessible after conventional adaptive immune targeting has failed.

Engineering refinements have strengthened this platform. Directed evolution of NKp30 has generated variants with improved binding properties and functional performance [40], while CRISPR/Cas9-edited, TCR-deleted NKp30 CAR-T strategies have shown preclinical anti-melanoma activity [32]. Together, these advances move the field beyond initial proof-of-concept toward more refined engineered cell products.

At the same time, these strategies remain subject to the core biological constraints of the axis: heterogeneous expression, soluble ligand generation, and target instability under treatment pressure. The success of NKp30-based CAR approaches will likely depend on both receptor engineering and control of target biology.

## 7. Combination Strategies and Clinical Positioning

One obvious direction is combination with immune checkpoint blockade. In principle, B7-H6-directed cellular therapies or engagers could complement checkpoint inhibitors by broadening immune attack beyond classical T-cell-restricted recognition. This may be most useful in checkpoint-resistant, HLA-low, or dedifferentiated melanoma states that remain accessible through stress-linked surface targets such as B7-H6.

Combination with stress-inducing or target-inducing therapies is another attractive strategy. Experimental studies have shown that conventional anticancer stressors, including chemotherapy, radiotherapy, heat shock, and inflammatory cytokine exposure, as well as integrated stress response pathways, can increase B7-H6 expression [16,27]. This may be particularly relevant for cell-based or engager-based strategies, whose efficacy could depend on transient increases in membrane B7-H6 under treatment-related stress.

However, not all therapeutic combinations are likely to be favorable. Histone deacetylase inhibitors, for example, have been shown to downregulate B7-H6 through Histone deacetylase 2/3 (HDAC2/3)-dependent mechanisms, leading to impaired NK-cell recognition [41].

**Figure 2:**
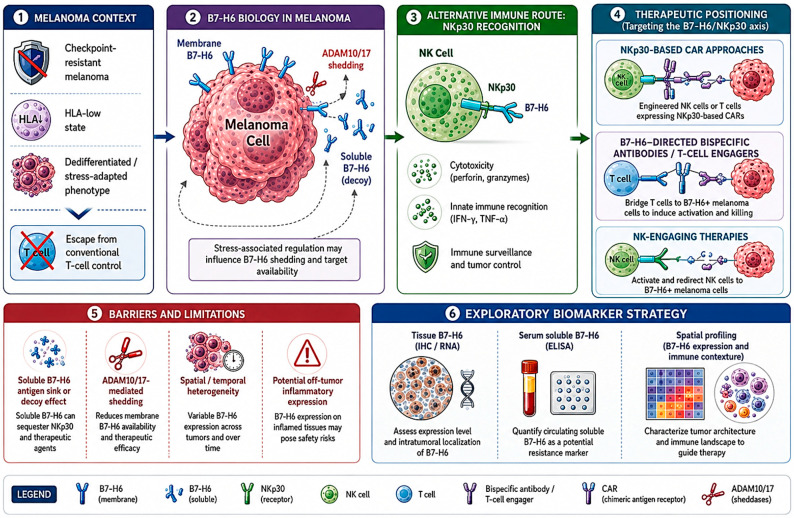
**Proposed therapeutic positioning of the B7-H6/NKp30 axis in checkpoint-resistant and HLA-low melanoma.** Checkpoint-resistant, HLA-low, dedifferentiated, or stress-adapted melanoma may become less accessible to conventional T-cell-mediated immune control while retaining potential susceptibility to NKp30-mediated recognition through membrane-bound B7-H6. B7-H6-directed strategies, including NKp30-based CAR approaches, bispecific antibodies/T-cell engagers, and NK-engaging therapies, may provide alternative immune-targeting opportunities. However, therapeutic development must account for ADAM10/17-mediated shedding, soluble B7-H6 antigen sink or decoy effects, spatial and temporal heterogeneity, and potential off-tumor expression under inflammatory conditions. An exploratory biomarker strategy integrating tissue B7-H6, serum soluble B7-H6, and spatial profiling may help define target accessibility, resistance mechanisms, and appropriate disease contexts for future translational studies. **Abbreviations:** ADAM10/17, A disintegrin and metalloproteinase domain-containing proteins 10 and 17; B7-H6, B7 homolog 6; CAR, chimeric antigen receptor; HLA, human leukocyte antigen; NKp30, natural cytotoxicity receptor 3; HLA, human leukocyte antigen; IHC, immunohistochemistry; IFN-γ, interferon gamma; TNF-α, tumor necrosis factor alpha. Arrows indicate proposed directional relationships among melanoma context, B7-H6 biology, immune recognition, therapeutic strategies, barriers, and biomarker assessment.

These considerations argue for dynamic target assessment, including serial evaluation of tissue and soluble B7-H6 when feasible, rather than relying solely on baseline positivity. Clinically, B7-H6 is not yet a validated predictive biomarker or an established therapeutic class in melanoma. However, the strongest near-term opportunity is likely to involve biologically enriched settings, particularly checkpoint-resistant, HLA-low, dedifferentiated, or therapy-adapted melanoma states, in which stress-linked surface vulnerabilities may remain accessible despite failure of conventional immune control. Figure 2 summarizes a proposed translational framework for positioning the B7-H6/NKp30 axis in checkpoint-resistant, HLA-low, dedifferentiated, or stress-adapted melanoma states, integrating therapeutic opportunities with key biologic and biomarker limitations.

In that context, B7-H6 warrants focused translational study as both a target and a state-linked biomarker.

## 8. Conclusions

The B7-H6/NKp30 axis sits at the intersection of immune surveillance, tumor escape, and therapeutic development in melanoma. Initially described as a mechanism of NK-cell recognition, this pathway now has broader relevance: B7-H6 is linked to membrane targetability, soluble biomarker potential, protease-mediated escape, and emerging tumor-intrinsic functions related to survival and migration.

At the same time, the biology of the axis argues against overly simplistic interpretation. In melanoma, B7-H6 appears heterogeneous, dynamically regulated, and influenced by target form, shedding, and treatment pressure. Accordingly, this pathway is best viewed not as a static antigenic marker, but as a context-dependent indicator of targetable tumor state.

A major strength of the axis is its translational flexibility across bispecific, NK-engaging, and NKp30-based CAR platforms. This breadth supports therapeutic development in melanoma settings where conventional immune control is limited, including immune-excluded, antigen-presentation-defective, and therapy-adapted states.

The key challenge now is prioritization. Future work should define where B7-H6 is most meaningfully expressed in melanoma, how membrane-bound and soluble forms should be interpreted together, and which disease states are most suitable for early clinical translation. The strongest near-term opportunity may lie not in unselected melanoma, but in biologically enriched settings such as checkpoint-resistant, HLA-low, dedifferentiated, or stress-adapted disease, where stress-linked surface vulnerabilities may remain actionable. In that context, the B7-H6/NKp30 axis warrants focused translational study as both a therapeutic target and a dynamic biomarker.

## Figures and Tables

**Table 1:** Melanoma-Relevant Evidence Supporting Investigation of the B7-H6/NKp30 Axis. (**A**). Preclinical and mechanistic evidence. (**B**). Clinical and patient-sample evidence.

(**A**)					
**Study**	**Model or Evidence Type**	**Key Melanoma-Linked Observation**	**Translational Implication**	**Main Limitation**	
Brandt et al. (2009) [3]	Discovery study; tumor cell-line expression analysis	Identified B7-H6 as the human tumor-cell ligand for NKp30; melanoma cell lines were included among tumor types reported to express B7-H6.	Establishes biologic plausibility for melanoma as a B7-H6-positive tumor context.	Discovery-phase evidence; limited melanoma-specific clinical annotation and no therapeutic validation in melanoma.	
Schlecker et al. (2014) [7]	Mechanistic shedding study	Demonstrated ADAM10/17-mediated shedding of B7-H6, reducing surface ligand availability and generating soluble B7-H6.	Provides a mechanistic basis for target loss, soluble biomarker development, and resistance to surface-directed strategies.	ADAM10/17 inhibition is mechanistically informative but not yet a clinically realistic melanoma strategy.	
Obiedat et al. (2020) [16]	Stress-response regulation	Showed that integrated stress response signaling can increase B7-H6 expression.	Supports the concept that B7-H6 may mark stress-adapted tumor states and could be dynamically regulated by therapy-related stress.	Not melanoma-specific; relevance to melanoma requires direct validation.	
Mohammadi et al. (2023) [14]	A375 melanoma cell-line study	B7-H6 silencing reduced melanoma cell survival, migration, and clonogenicity and increased dacarbazine sensitivity.	Suggests that B7-H6-positive melanoma states may have tumor-intrinsic relevance beyond NK-cell recognition.	Single-cell-line study; no in vivo melanoma validation and no patient-level correlation.	
Givi et al. (2025) [32]	Primary melanoma samples, melanoma cell lines, and A375 xenograft model	Reported B7-H6 expression in primary melanoma samples and melanoma cell lines; NKp30 CAR TCRKO cells showed antitumor activity against A375 melanoma in vitro and in vivo.	Provides the strongest direct preclinical support for B7-H6-directed cellular therapy in melanoma.	Small melanoma sample set; in vivo testing relied on an A375 xenograft model; no clinical melanoma efficacy data.	
(**B**)					
**Study**	**Patient or Specimen Number**	**Sample Type**	**B7-H6/NKp30 Feature Assessed**	**Key Finding**	**Main Limitation**
Schlecker et al. (2014) [7]	Melanoma tissue: stage III *n* = 21, stage IV *n* = 19; serum: melanoma *n* = 93, healthy donors *n* = 32; paired tissue/serum assessment in *n* = 3 stage IV cases	Melanoma tissue and serum	B7-H6 mRNA, soluble B7-H6, and tissue B7-H6 expression	Soluble B7-H6 was elevated in a subset of melanoma sera, and tissue B7-H6 was detectable in selected melanoma specimens.	No outcome-linked validation; limited paired tissue-serum analysis; does not define spatial heterogeneity, treatment response, or predictive value.
Messaoudene et al. (2016) [21]	Metastatic melanoma patient cohort; reported blood NKp30 isoform/NKp46 transcript profiling	Peripheral blood	NKp30 isoforms and NKp46 transcripts	Supports the clinical relevance of NK-cell receptor biology in melanoma progression and outcome.	Does not directly assess tumor-cell B7-H6 expression or B7-H6-directed therapy.
Givi et al. (2025) [32]	Primary melanoma samples *n* = 3; melanoma cell lines also assessed	Patient-derived melanoma samples and melanoma cell lines	B7-H6 mRNA and cell-surface protein expression	B7-H6 was detected in patient-derived melanoma samples and melanoma cell lines, with evidence of variable transcript/protein relationships.	Very small patient-sample set; no primary/metastatic cohort analysis, spatial profiling, longitudinal sampling, or clinical outcome association.

**Abbreviations:** ADAM10/17, A disintegrin and metalloproteinase domain-containing proteins 10 and 17; CAR-T, chimeric antigen receptor T-cell therapy; HLA, human leukocyte antigen; NK, natural killer; ADAM10/17, A disintegrin and metalloproteinase domain-containing proteins 10 and 17; B7-H6, B7 homolog 6; CAR, chimeric antigen receptor; NK, natural killer; NKp30, natural cytotoxicity receptor 3; NKp46, natural cytotoxicity receptor 1; TCRKO, T-cell receptor knockout.

**Table 2:** Therapeutic Strategies Targeting B7-H6/NKp30 and Their Development Status for Melanoma. (**A**). Preclinical therapeutic strategies. (**B**). Clinical-development status.

(**A**)					
**Platform**	**Key Reference**	**Mechanistic Concept**	**Melanoma-Specific Evidence**	**Melanoma Patients Treated or Reported**	**Main Development Caveat**
NKp30-based CAR-T cells	Zhang et al. (2012) [36]	Uses the extracellular domain of NKp30 to redirect T cells against B7-H6-positive tumor cells.	Provides proof-of-concept for NKp30 CAR recognition of B7-H6-positive tumors, but not melanoma-specific.	0	Preclinical platform; target density, soluble B7-H6, and melanoma heterogeneity were not addressed.
B7-H6-specific CAR-T cells	Wu et al. (2015) [39]	Uses a B7-H6-specific CAR design to target B7-H6-positive tumor cells.	Demonstrates CAR feasibility against B7-H6-positive tumors, but melanoma-specific validation remains limited.	0	Preclinical evidence; melanoma-specific efficacy, safety, antigen-density requirements, and resistance mechanisms remain undefined.
CRISPR/Cas9 TCR-edited NKp30 CAR-T cells	Givi et al. (2025) [32]	Combines NKp30-based CAR recognition with TCR deletion to generate allogeneic NKp30 CAR TCRKO cells.	Direct melanoma evidence in A375 in vitro and A375 NSG xenograft models; primary melanoma samples were assessed for B7-H6 expression.	0	Strongest melanoma-directed preclinical therapeutic evidence, but still limited to small sample numbers and xenograft modeling.
B7-H6/CD3 bispecific T-cell engagers	Wu et al. (2015) [37]	Redirects CD3-positive T cells toward B7-H6-positive tumor cells.	Supports druggability of B7-H6 through T-cell redirection, but not melanoma-specific.	0	Preclinical evidence; melanoma-specific efficacy, safety, and resistance mechanisms remain undefined.
B7-H6/CD3 IgG-like T-cell engager BI 765049	Zhang et al. (2022) [15]	IgG-like bispecific antibody engaging B7-H6 on tumor cells and CD3 on T cells.	Public preclinical data are strongest in gastrointestinal tumor models, not melanoma.	0 in melanoma-specific published data	Supports clinical tractability of the target, but does not validate B7-H6-directed therapy in melanoma.
Affinity-matured B7-H6/NKp30 immunoligands	Pekar et al. (2021) [38]	Enhances NK-cell engagement through optimized B7-H6/NKp30 interaction.	No melanoma-specific therapeutic validation.	0	May improve activity in low-density target settings, but melanoma relevance remains extrapolated.
Dual T/NK engagement plus localized IL-15/IL-15Rα signaling	Ma et al. (2025) [35]	Combines B7-H6-targeted immune redirection with cytokine-supported effector-cell activation.	Not melanoma-specific.	0	Cytokine dosing, safety, and generalizability to melanoma remain unresolved.
Shedding-aware strategies	Schlecker et al. (2014) [7]	Attempts to preserve membrane B7-H6 by limiting ADAM10/17-mediated shedding.	Mechanistically relevant because soluble B7-H6 has been detected in melanoma serum.	0	ADAM10/17 inhibition is not yet a realistic clinical melanoma strategy; soluble B7-H6 may still limit CAR or engager efficacy.
(**B**)					
**Agent or Strategy**	**Clinical-Development Context**	**Melanoma-Specific Clinical Data**	**Melanoma Patients Treated or Reported**	**Interpretation for Melanoma**	
BI 765049, B7-H6/CD3 T-cell engager, with or without ezabenlimab	Phase I/early clinical testing in advanced B7-H6-positive solid tumors [15]	No melanoma-specific response or outcome data reported in the cited melanoma-focused literature.	Not reported as melanoma-specific	Demonstrates that B7-H6 is being pursued clinically, but it does not establish B7-H6 as a validated melanoma target.	
B7-H6-directed CAR-T or NKp30 CAR-T therapy	Preclinical cellular therapy development	No clinical melanoma cohort reported in the reviewed literature.	0	Melanoma translation remains preclinical and should be framed as investigational.	
B7-H6/NKp30 as a biomarker-guided therapeutic strategy	Proposed tissue plus soluble B7-H6 assessment	No prospective melanoma trial has validated B7-H6 selection, soluble B7-H6 monitoring, or membrane B7-H6 density as predictive biomarkers.	0	Future trials should incorporate membrane target density, soluble B7-H6, spatial heterogeneity, and NK-cell competence before patient selection can be justified.	

**Abbreviations:** ADAM10/17, A disintegrin and metalloproteinase domain-containing proteins 10 and 17; CAR-T, chimeric antigen receptor T-cell therapy; CD3, cluster of differentiation 3; IL-15, interleukin-15; NK, natural killer; TCR, T-cell receptor.

## Data Availability

No new data were created or analyzed in this study. Data sharing is not applicable to this article.

## Abbreviations

The following abbreviations are used in this manuscript:

**B7-H6**	B7 homolog 6
**NKp30**	Natural cytotoxicity receptor 3 (NCR3)
**ADAM10**	A disintegrin and metalloproteinase domain-containing protein 10
**ADAM17**	A disintegrin and metalloproteinase domain-containing protein 17
**CAR-T**	Chimeric antigenic receptor T-cell therapy
**c-Myc**	Cellular homolog of v-Myc oncogene
**PERK**	Protein kinase R (PKR)-like endoplasmic reticulum kinase
**CRISPR/Cas9**	Clustered regularly interspaced short palindromic repeat/CRISPR-associated protein 9
**HDAC2/3**	Histone deacetylase 2/3

## References

[1] Haugh A.M., Salama A.K.S., Johnson D.B. (2021). Advanced Melanoma: Resistance Mechanisms to Current Therapies. Hematol. Oncol. Clin. N. Am..

[2] Fenton S.E., Sosman J.A., Chandra S. (2019). Resistance mechanisms in melanoma to immuneoncologic therapy with checkpoint inhibitors. Cancer Drug Resist..

[3] Brandt C.S., Baratin M., Yi E.C., Kennedy J., Gao Z., Fox B., Haldeman B., Ostrander C.D., Kaifu T., Chabannon C. (2009). The B7 family member B7-H6 is a tumor cell ligand for the activating natural killer cell receptor NKp30 in humans. J. Exp. Med..

[4] Li Y., Wang Q., Mariuzza R.A. (2011). Structure of the human activating natural cytotoxicity receptor NKp30 bound to its tumor cell ligand B7-H6. J. Exp. Med..

[5] Pinheiro P.F., Justino G.C., Marques M.M. (2020). NKp30—A prospective target for new cancer immunotherapy strategies. Br. J. Pharmacol..

[6] Kaifu T., Escalière B., Gastinel L.N., Vivier E., Baratin M. (2011). B7-H6/NKp30 interaction: A mechanism of alerting NK cells against tumors. Cell Mol. Life Sci..

[7] Schlecker E., Fiegler N., Arnold A., Altevogt P., Rose-John S., Moldenhauer G., Sucker A., Paschen A., von Strandmann E.P., Textor S. (2014). Metalloprotease-mediated tumor cell shedding of B7-H6, the ligand of the natural killer cell-activating receptor NKp30. Cancer Res..

[8] Kageshita T., Hirai S., Ono T., Hicklin D.J., Ferrone S. (1999). Down-regulation of HLA class I antigen-processing molecules in malignant melanoma: Association with disease progression. Am. J. Pathol..

[9] Rambow F., Marine J.C., Goding C.R. (2019). Melanoma plasticity and phenotypic diversity: Therapeutic barriers and opportunities. Genes. Dev..

[10] Lee J.H., Shklovskaya E., Lim S.Y., Carlino M.S., Menzies A.M., Stewart A., Pedersen B., Irvine M., Alavi S., Yang J.Y.H. (2020). Transcriptional downregulation of MHC class I and melanoma de- differentiation in resistance to PD-1 inhibition. Nat. Commun..

[11] Sottile R., Pangigadde P.N., Tan T., Anichini A., Sabbatino F., Trecroci F., Favoino E., Orgiano L., Roberts J., Ferrone S. (2016). HLA class I downregulation is associated with enhanced NK-cell killing of melanoma cells with acquired drug resistance to BRAF inhibitors. Eur. J. Immunol..

[12] Lehmann J., Caduff N., Krzywińska E., Stierli S., Salas-Bastos A., Loos B., Levesque M.P., Dummer R., Stockmann C., Münz C. (2023). Escape from NK cell tumor surveillance by NGFR-induced lipid remodeling in melanoma. Sci. Adv..

[13] Porgador A., Mandelboim O., Restifo N.P., Strominger J.L. (1997). Natural killer cell lines kill autologous beta2-microglobulin-deficient melanoma cells: Implications for cancer immunotherapy. Proc. Natl. Acad. Sci. USA.

[14] Mohammadi A., Najafi S., Amini M., Baradaran B., Firouzamandi M. (2023). B7H6 silencing increases chemosensitivity to dacarbazine and suppresses cell survival and migration in cutaneous melanoma. Melanoma Res..

[15] Zhang W., Auguste A., Liao X., Walterskirchen C., Bauer K., Lin Y.H., Yang L., Sayedian F., Fabits M., Bergmann M. (2022). A Novel B7-H6-Targeted IgG-Like T Cell-Engaging Antibody for the Treatment of Gastrointestinal Tumors. Clin. Cancer Res..

[16] Obiedat A., Charpak-Amikam Y., Tai-Schmiedel J., Seidel E., Mahameed M., Avril T., Stern-Ginossar N., Springuel L., Bolsée J., Gilham D.E. (2020). The integrated stress response promotes B7H6 expression. J. Mol. Med..

[17] Laskowski T.J., Biederstädt A., Rezvani K. (2022). Natural killer cells in antitumour adoptive cell immunotherapy. Nat. Rev. Cancer.

[18] Lanier L.L. (2005). NK cell recognition. Annu. Rev. Immunol..

[19] Kärre K. (2008). Natural killer cell recognition of missing self. Nat. Immunol..

[20] Morgado S., Sanchez-Correa B., Casado J.G., Duran E., Gayoso I., Labella F., Solana R., Tarazona R. (2011). NK cell recognition and killing of melanoma cells is controlled by multiple activating receptor-ligand interactions. J. Innate Immun..

[21] Messaoudene M., Fregni G., Enot D., Jacquelot N., Neves E., Germaud N., Garchon H.J., Boukouaci W., Tamouza R., Chanal J. (2016). NKp30 isoforms and NKp46 transcripts in metastatic melanoma patients: Unique NKp30 pattern in rare melanoma patients with favorable evolution. Oncoimmunology.

[22] Balsamo M., Scordamaglia F., Pietra G., Manzini C., Cantoni C., Boitano M., Queirolo P., Vermi W., Facchetti F., Moretta A. (2009). Melanoma-associated fibroblasts modulate NK cell phenotype and antitumor cytotoxicity. Proc. Natl. Acad. Sci. USA.

[23] Jilani S., Saco J.D., Mugarza E., Pujol-Morcillo A., Chokry J., Ng C., Abril-Rodriguez G., Berger-Manerio D., Pant A., Hu J. (2024). CAR-T cell therapy targeting surface expression of TYRP1 to treat cutaneous and rare melanoma subtypes. Nat. Commun..

[24] Anikeeva N., Panteleev S., Mazzanti N.W., Terai M., Sato T., Sykulev Y. (2021). Efficient killing of tumor cells by CAR-T cells requires greater number of engaged CARs than TCRs. J. Biol. Chem..

[25] Ponath V., Hoffmann N., Bergmann L., Mäder C., Alashkar Alhamwe B., Preußer C., Pogge von Strandmann E. (2021). Secreted Ligands of the NK Cell Receptor NKp30: B7-H6 Is in Contrast to BAG6 Only Marginally Released via Extracellular Vesicles. Int. J. Mol. Sci..

[26] Textor S., Bossler F., Henrich K.O., Gartlgruber M., Pollmann J., Fiegler N., Arnold A., Westermann F., Waldburger N., Breuhahn K. (2016). The proto-oncogene Myc drives expression of the NK cell-activating NKp30 ligand B7-H6 in tumor cells. Oncoimmunology.

[27] Cao G., Wang J., Zheng X., Wei H., Tian Z., Sun R. (2015). Tumor Therapeutics Work as Stress Inducers to Enhance Tumor Sensitivity to Natural Killer (NK) Cell Cytolysis by Up-regulating NKp30 Ligand B7-H6. J. Biol. Chem..

[28] Chen H., Zhang Y., Shen Y., Jiang L., Zhang G., Zhang X., Xu Y., Fu F. (2023). Deficiency of N-linked glycosylation impairs immune function of B7-H6. Front. Immunol..

[29] Vendramin R., Katopodi V., Cinque S., Konnova A., Knezevic Z., Adnane S., Verheyden Y., Karras P., Demesmaeker E., Bosisio F.M. (2021). Activation of the integrated stress response confers vulnerability to mitoribosome-targeting antibiotics in melanoma. J. Exp. Med..

[30] Čaval T., Alisson-Silva F., Schwarz F. (2023). Roles of glycosylation at the cancer cell surface: Opportunities for large scale glycoproteomics. Theranostics.

[31] Rodriguez E. (2025). Tumor Glycosylation: A Main Player in the Modulation of Immune Responses. Eur. J. Immunol..

[32] Givi S., Lohnes B.J., Ebrahimi S., Riedel S., Khokhali S., Khan S.A., Keller M., Wölfel C., Echchannaoui H., Bockamp E. (2025). CRISPR/Cas9 TCR-Edited NKp30 CAR T Cells Exhibit Superior Anti-Tumor Immunity to B7H6-Expressing Leukemia and Melanoma. Int. J. Mol. Sci..

[33] Reuben A., Spencer C.N., Prieto P.A., Gopalakrishnan V., Reddy S.M., Miller J.P., Mao X., De Macedo M.P., Chen J., Song X. (2017). Genomic and immune heterogeneity are associated with differential responses to therapy in melanoma. npj Genom. Med..

[34] Rusakiewicz S., Perier A., Semeraro M., Pitt J.M., Pogge von Strandmann E., Reiners K.S., Aspeslagh S., Pipéroglou C., Vély F., Ivagnes A. (2017). NKp30 isoforms and NKp30 ligands are predictive biomarkers of response to imatinib mesylate in metastatic GIST patients. Oncoimmunology.

[35] Ma X., He H., Zhu Y., Zuo D., Wang F., Feng M., Ji K., Chen X. (2025). Dual T/NK cell engagement via B7-H6-targeted bispecific antibodies and IL-15 eradicates chemo-resistant solid tumors. Front. Immunol..

[36] Zhang T., Wu M.R., Sentman C.L. (2012). An NKp30-based chimeric antigen receptor promotes T cell effector functions and antitumor efficacy in vivo. J. Immunol..

[37] Wu M.R., Zhang T., Gacerez A.T., Coupet T.A., DeMars L.R., Sentman C.L. (2015). B7H6-Specific Bispecific T Cell Engagers Lead to Tumor Elimination and Host Antitumor Immunity. J. Immunol..

[38] Pekar L., Klausz K., Busch M., Valldorf B., Kolmar H., Wesch D., Oberg H.H., Krohn S., Boje A.S., Gehlert C.L. (2021). Affinity Maturation of B7-H6 Translates into Enhanced NK Cell-Mediated Tumor Cell Lysis and Improved Proinflammatory Cytokine Release of Bispecific Immunoligands via NKp30 Engagement. J. Immunol..

[39] Wu M.R., Zhang T., DeMars L.R., Sentman C.L. (2015). B7H6-specific chimeric antigen receptors lead to tumor elimination and host antitumor immunity. Gene Ther..

[40] Butler S.E., Brog R.A., Chang C.H., Sentman C.L., Huang Y.H., Ackerman M.E. (2022). Engineering a natural ligand-based CAR: Directed evolution of the stress-receptor NKp30. Cancer Immunol. Immunother..

[41] Fiegler N., Textor S., Arnold A., Rölle A., Oehme I., Breuhahn K., Moldenhauer G., Witzens-Harig M., Cerwenka A. (2013). Downregulation of the activating NKp30 ligand B7-H6 by HDAC inhibitors impairs tumor cell recognition by NK cells. Blood.

